# Characterisation of secretome-based immune responses of human leukocytes infected with various *Mycobacterium tuberculosis* lineages

**DOI:** 10.7717/peerj.11565

**Published:** 2021-06-03

**Authors:** Benjawan Kaewseekhao, Sittiruk Roytrakul, Yodying Yingchutrakul, Marut Laohaviroj, Kanin Salao, Kiatichai Faksri

**Affiliations:** 1 Department of Microbiology, Faculty of Medicine, Khon Kaen University, Khon Kaen, Thailand; 2 Research and Diagnostic Center for Emerging Infectious Diseases (RCEID), Faculty of Medicine, Khon Kaen University, Khon Kaen, Thailand; 3 National Center for Genetic Engineering and Biotechnology (BIOTEC), National Science and Technology Development Agency (NSTDA), Pathumthani, Thailand

**Keywords:** East-Asian lineage, Euro-American lineage, Indo-Oceanic lineage, *Mycobacterium tuberculosis*, Proteomics

## Abstract

**Background:**

Differences in immune responses against different lineages of *Mycobacterium tuberculosis* (*Mtb*), and by different types of immune cell, are still poorly understood. We aimed to compare the secretome-based immune responses among three *Mtb* lineages and among immune-cell types. The immune responses were also investigated during infection and when the bacilli had been eliminated from the immune cells.

**Methods:**

Human primary leukocytes were infected with strains representing three lineages of *Mtb* (East-Asian, Indo-Oceanic and Euro-American). Label-free GeLC MS/MS proteomic analysis of secretomes was performed. The response of each immune-cell type was compared with the appropriate interactome database for each.

**Results:**

The expression pattern of proteins secreted by *Mtb*-infected leukocytes differed among *Mtb* lineages. The ancestral lineage (IO lineage) had a greater ability to activate MMP14 (associated with leukocyte migration) than did the more recent lineages (EA and EuA). During infection, proteins secreted by macrophages, dendritic cells, neutrophils and B-cells were associated with cell proliferation. Following clearance of *Mtb*, proteins associated with interferon signaling were found in macrophages, dendritic cells and neutrophils: proteins associated with antigen processing were found in B-cells and regulatory T-cells. Expression of immune response-related proteins from many immune-cell types might be suppressed by *Mtb* infection*.* Our study has provided a better insight into the host-pathogen interaction and immune response against different *Mtb* lineages.

## Introduction

Tuberculosis (TB) is the most important infectious disease globally, causing 10 million new cases and causing 1.5 million deaths annually (WHO, 2019). The only available vaccine is the Bacillus Calmette–Guérin (BCG) vaccine that can protect only against severe forms of TB in young children (Monteiro-Maia & Pinho, 2014). With the advance of research technology, knowledge regarding immune responses against *Mycobacterium tuberculosis* (*Mtb*) is progressing (Chai et al., 2020; De Martino et al., 2019). However, several gaps still remain. For example, it remains uncertain whether the immune response varies according to the lineage of *Mtb* involved, or according to the type of immune cell involved in the host response.

Major members of the *Mtb* complex have been classified, based on large sequence polymorphisms, into seven lineages that are associated with geographical regions: Indo-Oceanic (IO), East Asian (EA, including Beijing), East African Indian (EAI), Euro-American (EuA), West African1, West African2 and Ethiopian (Coscolla & Gagneux, 2014; Gagneux et al., 2006). EA, EuA and IO lineages of *Mtb* are commonly found in Southeast Asia (Gagneux et al., 2006). The *Mtb* lineages differ in virulence and transmissibility (Brites & Gagneux, 2012; Coscolla & Gagneux, 2014; Tientcheu et al., 2017). There have been few studies comparing immune responses against different *Mtb* strains using high-throughput analysis approaches (Li et al., 2017; Subbian et al., 2013). A transcriptomic study of rabbits infected with HN878 (a hyper-virulent strain of the EA lineage) and with CDC1551 (a hyper-immunogenic strain of the EuA lineage) found that the former induced higher gene expression of genes associated with macrophage activation as well as greater recruitment and activation of polymorphonuclear leukocytes (PMN) (Subbian et al., 2013). A proteomic study of a human monocytic cell line (THP-1) infected with H37Rv or H37Ra strains showed that H37Rv induced higher levels of expression of proteins associated with coagulations, inflammatory response and apoptosis, as well as oxidative phosphorylation (Li et al., 2017). However, no previous study has compared the immune responses mounted against the three main *Mtb* lineages (IO, EA, EuA).

Various host immune cells cooperate during immune responses against *Mtb* infection. Given that the pathogen is intracellular, macrophages and T-cells have been suggested as the major immune cells operating against *Mtb* infection (De Martino et al., 2019). Using classical cell-sorting techniques, the responses of particular immune cells, such as macrophages (Yuan et al., 2019) and T-cells (Yang et al., 2015), to *Mtb* infection have been demonstrated. However, the selective culture of particular types of immune cells diminishes our ability to observe interactions among these types. Using high-throughput techniques, the function of neutrophils during the *Mtb* infection process has been clarified (Gideon et al., 2019). However, no previous study has used a proteomic approach and human primary leukocytes (a mixture of several types of immune cells) to demonstrate the interactions among immune-cell types during active infection and following clearance of *Mtb* from the cells.

In this study, we aimed to characterise and compare the immune responses of human primary leukocytes infected with *Mtb* lineages including IO, EA and EuA using LC-MS/MS analysis. The immune responses were also compared among immune-cell types using protein databases specific for each cell type. Comparisons were also made between the responses during the infected state and after Mtb has been cleared from the cells.

## Materials & Methods

### Bacterial culture and inoculum preparation

Three clinical isolates of *Mtb* were selected from our biobank (Research and Diagnostic Center for Emerging Infectious Diseases (RCEID), Faculty of Medicine, Khon Kaen University). These represented three lineages commonly found in Southeast Asia: EA (strain number 15177), IO (strain number 15561) and EuA (strain number 19477). The genotypic characteristics of these isolates are described in Table S1. IO represents the ancestral lineage and the other strains represent modern lineages of *Mtb*. Bacterial cell suspensions were prepared as described previously (Kaewseekhao et al., 2020). Briefly, all bacterial strains were cultured in Middlebrook 7H9 with OADC for 14 days. *Mtb* H37Rv was used as the reference control strain. The concentrations of *Mtb* cells were measured and adjusted to 0.5 McFarland standards. Any clumping of *Mtb* cell suspensions was broken up by passing cultures through a 26-gauge needle set.

### Infection experiment

Leukocytes, isolated from blood of three participants using HetaSep, were cultured in RPMI medium. The leukocyte culture from each host was used separately in three independent replicates of all experiments. All participants were healthy with an average age of 30.7 ± 3.1 years, Bacillus Calmette–Guérin (BCG) vaccinated and negative for TB according to the interferon gamma release assay (IGRA). The protocol used to infect leukocytes has been described previously (Kaewseekhao et al., 2020). Briefly, the leukocytes were activated by 50 nM phorbol myristate acetate (PMA) for 24 h then exposed to infection with *Mtb* (multiplicity of infection = 1) for 4 h, after which they were deemed to be infected. The *Mtb*-infected cells were then treated with a combination of isoniazid (INH: 3 µg/ml) and rifampicin (RIF: 9 µg/ml) starting 4 h after the start of exposure to *Mtb*. These drug concentrations were optimized for bacillary killing within 3 days (Kaewseekhao et al., 2015a). Medium containing the drugs was changed every 24 h. Uninfected cells treated with the combination of isoniazid and rifampicin were used as negative controls. The infection state at the beginning and the clearance state at the end of the experiments was confirmed using CFU counts on M7H11 plates, which were incubated for at least 4 weeks (Kaewseekhao et al., 2015b). Written informed consents were received from all participants. This study protocol (number HE581377) was approved by Ethics Committee in Human Research Ethics, Office of The Khon Kaen University.

### Extracellular protein collection and preparation

The extracellular proteins from each replicate (from each host) were collected by pipetting the extracellular fluids and mixing these with sodium dodecyl sulfate (SDS: final concentration 0.5% w/v). The intact infected leukocytes were discarded. The protocol of protein preparation has been described previously (Kaewseekhao et al., 2020). Briefly, protein concentrations were measured using the Lowry method (Waterborg, 2009). Bovine serum albumin (BSA) was used as a standard (0, 2, 4, 6, 8, 10 µg) for estimation of sample protein concentration. The BSA standard and sample were transferred into 96-well plates (performed in triplicate). Each sample was incubated with 200 µl of solution A (2.5% SDS, 2.5% Na_2_CO_3_, 0.2 N NaOH, 0.025% CuSO_4_ and 0.05% tartaric acid) at room temperature for 30 min. Then, 50 µl of solution B (20% Folin-Ciocalteu phenol reagent) was added and the mixture incubated at room temperature for 30 min. The protein concentrations were measured at OD750 nm and compared with the standard curve for BSA.

### SDS PAGE and in-gel digestion

The protein samples (100 µg from each of the three donors) were pooled and mixed by pipetting for 1 min. Then, 50 µg of the pooled protein was separated by SDS-PAGE and the gels were stained with Coomassie blue. In-gel digestion was done as described previously (Kaewseekhao et al., 2020). Briefly, the stained gel in each lane was cut into 11 pieces and then further cut into one mm^3^ cubes. The region of the gel with high molecular-weight proteins including BSA was excluded. All gel cubes from each sample were transferred into a 96-well plate and tryptic digestion was performed. The gel pieces were incubated with 25 mM NH_4_HCO_3_ for 10 min. Then, 200 µl acetonitrile (ACN) was added and the plate incubated for 10 min at room temperature. The supernatants were discarded. Then, 10 mM DTT in 10 mM NH_4_HCO_3_ was added and the plate incubated at 56 °C for 1 hr. Next, 100 mM iodoacetamide in 10 mM NH_4_HCO_3_ was added and incubated at room temperature for 1 hr in the dark. Then, 200 µl of ACN was added twice and all liquid was removed from each well. Tryptic digestion was performed by incubation of the gel pieces with 10 ng/µl trypsin in 10 mM NH_4_HCO_3_ at 37 °C for 3 h. The protein extraction was done by adding 50% ACN and shaking the plate at room temperature for 10 min for 3 cycles. Peptide solutions were dried at 40 °C and kept at −20 °C until analysis.

### LC-MS/MS analysis

LC-MS/MS analysis was done as previously described (Kaewseekhao et al., 2020). Briefly, the peptide samples were resuspended in 10 µl of 0.1% formic acid and transferred into low-protein-binding tubes. Large proteins were precipitated by centrifugation at 8,000×g for 10 min and the solution was transferred into vial tubes. Then, 4.5 µl of peptide sample was injected into a LC MS/MS analyser (hybrid quadrupole Q-TOF Impact II™, Bruker Daltonics). Separation of tryptic peptides was done using an Ultimate3000 Nano/Capillary LC System (Thermo Scientific, UK) coupled with a nano-CaptiveSpray™ ion source (Bruker Daltonics). Mobile phase A (0.1% formic acid) was used to transfer the samples at a flow rate of 15 µl/min for 1 min. Mobile phase B (5–50% solution of 0.1% formic acid in 80% acetonitrile) was used for solutions containing separated peptides with a flow rate of 600 nl/min for 15 min. Electrospray ionisation was carried out at 1.6 kV using the CaptiveSpray ion source. The column was rinsed with 80% mobile phase B for 3 min and column temperature was maintained at 35 °C. The MS/MS masses were screened over the range 150 to 2,200 Da and 0.5 s scan time. The LC MS/MS raw data have been deposited in the MassIVE database (https://massive.ucsd.edu) with accession No. MSV000084863.

### Bioinformatics and data analyses

LC-MS/MS data analysis was done as previously described (Kaewseekhao et al., 2020). Briefly, LC MS/MS raw data files (.mzXML) were analysed using DeCyderMS 2.0 differential analysis software (DeCyderMS, GE Healthcare Life Science, UK) and Mascot software (Matrix Science, London, UK) for protein identification based on the NCBI database. In brief, the taxonomy search specified human or eukaryote, enzyme (trypsin), variable modifications (carbamidomethyl, oxidation of methionine residues), mass values (monoisotopic), protein mass (unrestricted), peptide mass tolerance (± 1.2 Da), fragment mass tolerance (± 0.6 Da), peptide charge state (1+, 2+, and 3+), and max missed cleavages. Log2 expression levels of the proteins were calculated. Proteins induced by infection (any lineage of *Mtb*) were identified by disregarding those also expressed in uninfected controls.

### Protein network analysis

The detected proteins were classified into five groups according to their pattern of presence/ absence among conditions, i.e., whether they were upregulated or suppressed during initial infection (Day 1), sustained infection (Day 1 and Day 5), or following clearance from infected cells (Day 5 only). The criteria for this classification are described (Table S2). The candidate proteins in each group were used for protein network analysis. The union set of the leukocyte proteins was used for network analyses using NetworkAnalyst program (Xia, Benner & Hancock, 2014) based on entrez ID converted from GI number by db2db database. Network construction was done using IMEx Interactome and analysed by reference to the Reactome database (Orchard et al., 2012). The secretomes of leukocytes infected with each *Mtb* lineage were used for multi immune-cell type analyses based on the Immuno-Navigator database and the protein pathway analysis based on the Reactome database (Vandenbon et al., 2016). The expression levels of proteins in each group were plotted as heat maps using the ComplexHeatmap package in R.

## Results

### Overall proteomic response of primary leukocytes infected with *Mtb* and protein classification

**Figure 1:**
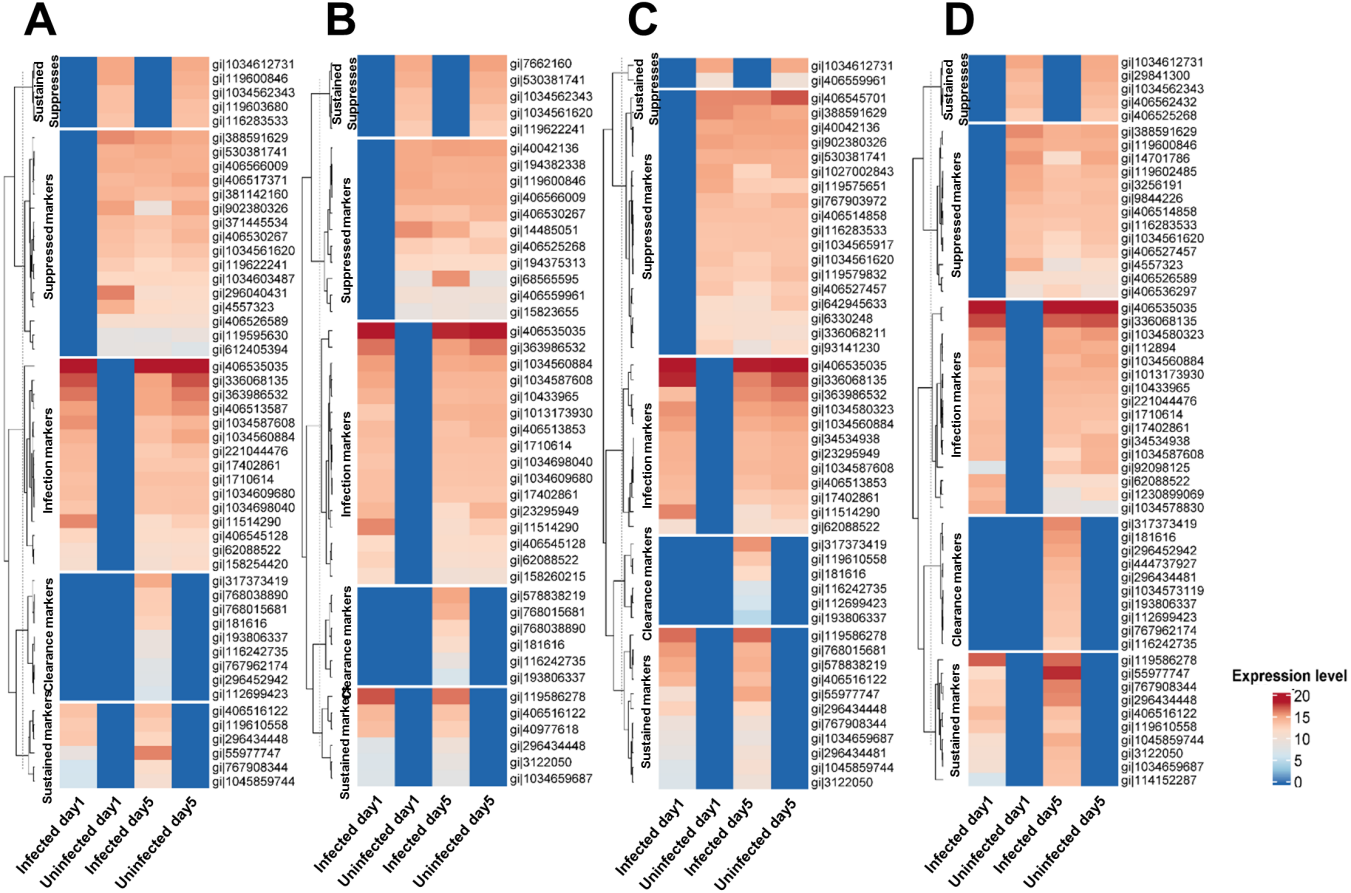
Classification of proteins secreted by primary leukocytes infected with different *M. tuberculosis* lineages. Five categories of protein markers were recognized among *Mtb* lineages: markers of initial infection and of sustained infection, markers suppressed during initial infection and during sustained infection, and markers differentially expressed after clearance of infection. Classification criteria are shown in Table S2. Proteins secreted by primary leukocytes infected with EA lineage (A). Proteins secreted by primary leukocytes infected with IO lineage (B). Proteins secreted by primary leukocytes infected with EUA lineage (C). Proteins secreted by primary leukocytes infected with H37Rv lineage (D). The elevated expression of some infection markers in control cells at Day 5 (compared to Day 1) could be due to cell aging and/or the background response to the long-term anti-TB drug treatment.

Secreted proteins were detected from leukocytes cells infected with the various *Mtb* lineages (union set; detected from at least one *Mtb* lineage) (*n* = 1,052) and following clearance of infection (*n* = 1,050). Based on the pattern of their expression among experimental conditions, these proteins were separated into five groups: markers of infection and of sustained infection, markers suppressed in infection and sustained infection, and markers of infection clearance (Fig. 1 and Table S3). These proteins were also classified according to the lineage of *Mtb* involved*.* The CFU plate count showed that *Mtb* cells were present in the leukocytes during the infection stage of the experiment but that no *Mtb* cells remained at the clearance stage (Day 5) (Table S4).

### Proteomics during infection and clearance states of leukocytes infected with *Mtb*

Using stringent criteria (Table S2), subsets of proteins expressed by leukocytes during infection or after clearance of infection with *Mtb* were identified (Table S3)*.* There were 35 proteins (based on GI number) exhibiting enhanced expression during infection with *Mtb* (Table S5). There were 67 proteins (based on GI number) suppressed at Day 1 or at Days 1 and 5 of *Mtb* infection (Table S5). Similarly, there were 42 (based on GI number) over-expressed following clearance of infection with *Mtb* (Table S5).

**Figure 2:**
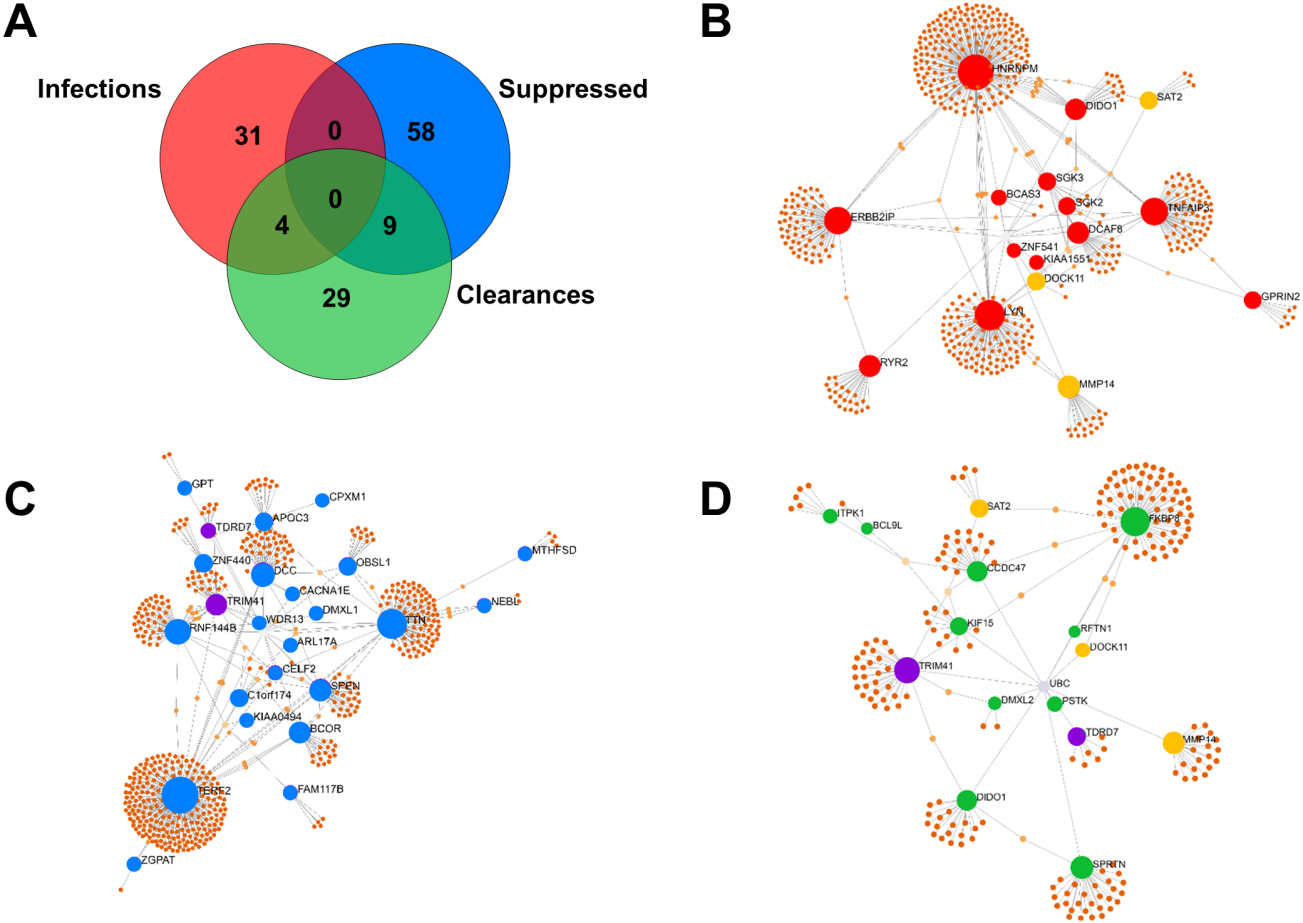
Comparison of proteins expressed in infection and clearance state of leukocytes infected with *M. tuberculosis* (union set among EA, IO and EUA linages and H37Rv control). The number of proteins (not found in the controls) detected during initial infection, clearance state of *Mtb* and proteins that were suppressed during initial infection (A). Network analysis of proteins enhanced in *Mtb*-infected cells (B). Network analysis of *Mtb*-suppressed proteins during initial infection (C). Network analysis of *Mtb*-clearance proteins (D). Red dots represent proteins expressed during the infection state with levels at least four-fold higher than in uninfected controls, yellow dots represent proteins expressed in both infection and clearance states, blue dots represent proteins suppressed during the infection state with levels at least four-fold lower than in uninfected controls, purple dots represent proteins expressed in both suppression and clearance states, green dots represent proteins expressed in the clearance state with levels at least four-fold higher than in uninfected controls and brown dots represent proteins linked to those proteins expressed in each condition.

Of the 35 *Mtb*-infection proteins, 16 had matches in the interactome database and were used for network analysis (Fig. 2B and Table S6). Of the 67 proteins suppressed by *Mtb* during initial infection, 24 had matches in the interactome database and were used for network analysis (Fig. 2C and Table S6). Of the 42 *Mtb*-clearance proteins, 15 had matches in the interactome database and were used for network analysis (Fig. 2D and Table S6). SAT2, DOCK11 and MMP14 were found in both infection and clearance states of *Mtb* (Figs. 2C and 2D) but were not present in control cells*.* TRD7 and TRIM41 were suppressed during initial infection and also found in the clearance states of *Mtb* (Figs. 2B and 2D). TRIM41 and RNF144B were the major *Mtb*-suppressed proteins. Expression of TRIM41 was 12.44-fold lower in H37Rv infection compared to uninfected controls. Expression levels of RNF144B were 14.35-fold lower in leukocytes infected with H37Rv, IO and EuA strains compared to uninfected controls (Table S3).

### Proteomic patterns from infected leukocytes differ according to the lineage of *Mtb* involved

**Figure 3:**
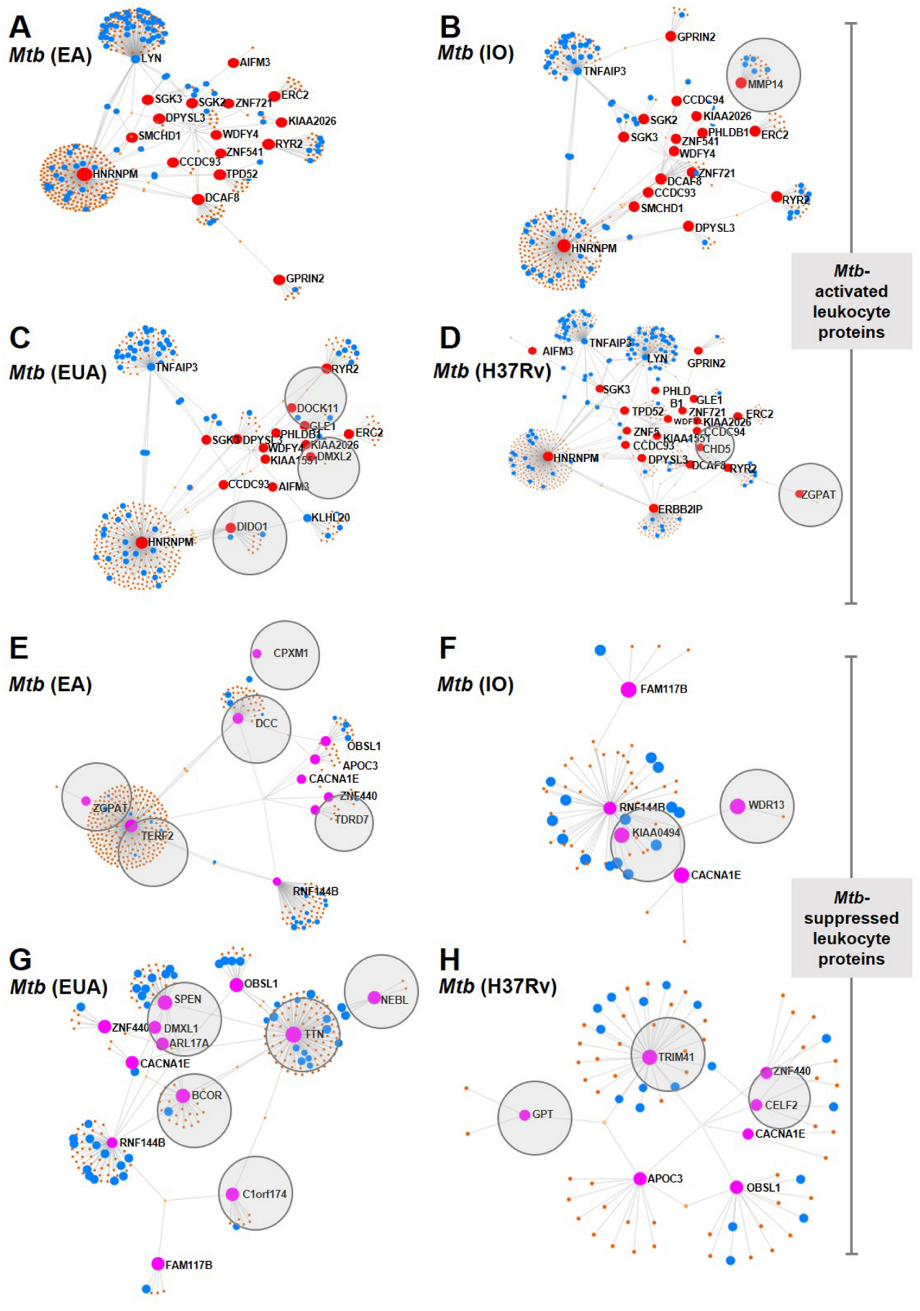
Network analysis of leukocyte proteins activated or suppressed by different lineages of *M. tuberculosis*. Network diagrams of *Mtb*-activated leukocyte proteins (present at Day 1 or (present at Day 1 and Day 5) (A–D) and of *Mtb*-suppressed leukocyte proteins (suppressed at Day 1 or (suppressed at Day 1 and Day 5) (E–H) compared among *Mtb* lineages. Protein networks of leukocytes infected with *Mtb* EA lineage are shown in Figs. 4A and 4E, IO lineage (B, F), EU lineage (C, G) and H37Rv strain (D, H). Red dots represent proteins expressed more than four-fold higher during infection compared to uninfected controls, blue dots represent proteins associated with the immune response, brown dots represent proteins linked to those expressed during infection and gray circles represent proteins uniquely expressed in response to a particular *Mtb* lineage.

Leukocyte proteins enhanced or suppressed by *Mtb* infection were compared among *Mtb* lineages (Fig. 3). Among the upregulated proteins, MMP14 was only found in IO infections (Fig. 3B) and DOCK11, DIDO1 and DMXL2 were only found in leukocytes infected with the EuA lineage (Fig. 3C). No protein was found exclusively in cells infected with the EA lineage (Fig. 3A). ZGPAT and CHD5 were found only in leukocytes with H37Rv strain infection (Fig. 3D). A list of proteins from these networks expressed during *Mtb* infection is provided (Table S7).

Proteins that were suppressed by infection differed among *Mtb* lineages. CPXM1, DCC, ZGPAT, TERF2 and TDRD7 were suppressed only in leukocytes infected with the EA lineage (Fig. 3E). WDR13 and KIAA0494 proteins were suppressed only in IO-lineage infections (Fig. 3F). SPEN, DMXL1, APL17A, BCOR TTN, NEBL and C1orf174 were suppressed only in EuA-lineage infections (Fig. 3G). GPT, TRIM41 and CELF2 were suppressed only in cells infected with the H37Rv strain (Fig. 3H). A list of suppressed proteins from these networks is provided (Table S8).

### Proteomic patterns differ among immune-cell types in response to *Mtb* infection

**Figure 4:**
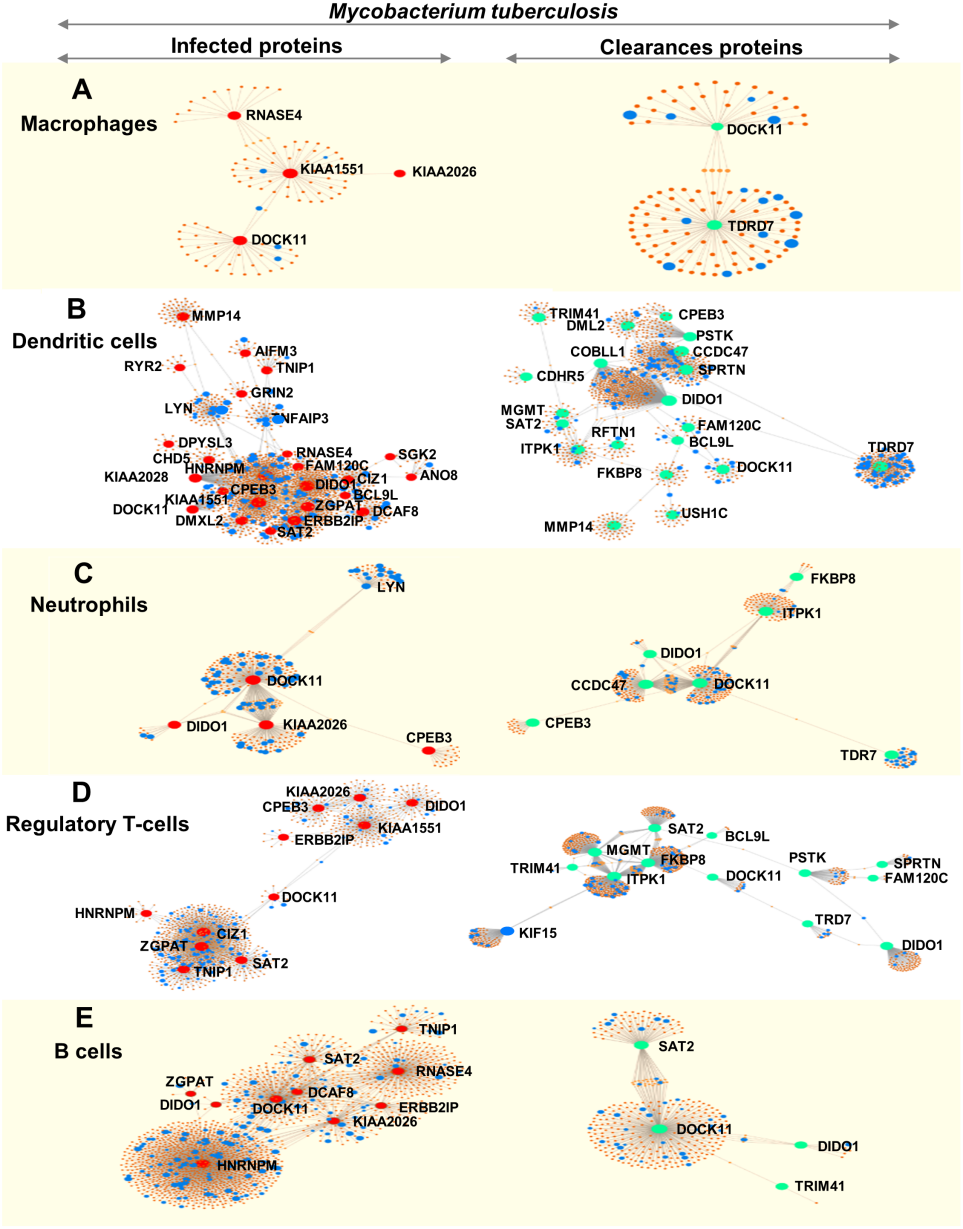
Networks of proteins secreted by leukocytes while infected with, and following clearance of, *M. tuberculosis* (any lineage) classified by immune-cell type (macrophages, dendritic cells, neutrophils, regulatory T-cells and B-cells). Networks are shown for macrophage-specific proteins (A), dendritic cell-specific proteins (B), neutrophil-specific proteins (C), regulatory T-cell-specific proteins (D) and B-cell-specific proteins (E). Red dots represent proteins expressed by infected leukocytes, green dots represent proteins expressed following clearance of infection, blue dots represent proteins associated with the immune response and orange-brown dots represent additional proteins complementing the pathways of those found in this study.

We analysed immune cell-specific molecules based on the Immuno-Navigator database of the secretome. Proteins specific to macrophages, dendritic cells, neutrophils, regulatory T-cells and B-cells were detected (Fig. 4).

During infection, macrophages secreted proteins involved in cell growth, mitosis and cell survival. Dendritic cells produced molecules involved in interleukin signaling, gene expression and cell-cycle progression. Neutrophils secreted proteins associated with signaling by interleukins and cell mitosis. Regulatory T-cells produced proteins involved in antigen processing and presentation to the immune system whereas proteins secreted by B-cells were associated with cell-cycle progression (Fig. 4 and Table S9).

Following clearance of infection, macrophages produced proteins involved in interferon-gamma signaling, phagocytosis and hemostasis. Proteins secreted by dendritic cells and neutrophils were involved in interferon signaling and cytokine signaling in the immune system. Regulatory T-cells secreted proteins involved in MHC class-II antigen presentation and cell-cycle progression, whereas B-cells produced proteins involved in cell-cycle activation and antigen presentation (peptide folding, assembly and loading of MHC class I) (Fig. 4 and Table S9).

## Discussion

In this study, we characterised and compared the secreted proteome of human primary leukocytes (containing mixed populations of immune cells) and infected with strains representing three major *Mtb* lineages: uninfected cells acted as controls. *Mtb* H37Rv, a widely studied laboratory stain with relatively low virulence compared to clinical isolates (Sarkar et al., 2012; Van Soolingen et al., 2004), was additionally used for comparisons with the clinical strains. The specific interactions of *Mtb* with the various host immune cell types are still unclear. We feel that study of the range of immune responses among the immune cell types during infection and following pathogen clearance from the host cells will provide insights into the mechanisms of host-pathogen interaction. We compared the three main lineages (EA, IO and EuA) of *Mtb* as these are commonly found in Southeast Asia. The EA lineage of *Mtb* is reportedly more virulent than other lineages (Lopez et al., 2003), and especially more than the ancestral IO lineage (Tram et al., 2018).

The overall immune-response proteins secreted by leukocytes infected with any lineage of *Mtb*, after disregarding those also secreted in similar quantities by in uninfected controls, were compared. We found that leukocytes infected with *Mtb* mainly secreted proteins involved in gene expression, translation and metabolism, reflecting the role of these cells in inducing an immune response against the pathogen. After *Mtb* was eliminated from the leukocytes, proteins from three main pathways—cellular protein degradation, antigen presentation and B-cell activation—were expressed. This is consistent with previous work from our group showing that clearance proteins from a monocytic cell line were associated with the cell cycle, RNA post-transcriptional modification, antimicrobial responses, cell proliferation, migration and movement (Kaewseekhao et al., 2015a). The current study used primary mixed leukocytes and found that most proteins secreted after clearance of *Mtb* and associated with the immune response belonged to two groups. First, those associated with cellular protein degradation and homeostasis, which might allow the leukocytes to adjust and return to a normal state following the challenge posed by infection. Second, proteins of the immune-response pathways, especially antigen presentation and B-cell activation, were found. This suggested that B-cells and the function of antigen presentation were initially suppressed during an active infection and were then restored after pathogen clearance. Alternatively, experience of infection might lead to trained immunity of innate cells (Lerm & Netea, 2016). This restored or trained function, and especially the ability to effectively process and present antigens to T-cells, is crucial to mount an adaptive immune response, and hence provide a long-term immune response to *Mtb* (Cooper, 2009). Further studies should be done to investigate functions of B-cells after the clearance of *Mtb* from the host. To better demonstrate possible trained immunity in cells that have experienced infection, other pathogen-killing methods, such as accelerated killing by immune cells themselves, might be used instead of anti-TB drugs.

*Mtb* has many immune-suppression strategies (De Martino et al., 2019; Zhai et al., 2019). We found that several immune-response pathways were suppressed by *Mtb* including MHC class I antigen processing and presentation, cytokine signaling, signaling by NGF and FCGR-dependent phagocytosis. Our study has provided more insight into these suppression strategies as follows. First, we found that tripartite motif 41 (TRIM41) was suppressed during *Mtb* infection. *Mtb* might suppress MHC class I antigen presentation via TRIM41. The TRIM protein family is involved in innate immune inflammatory responses and has the potential to be used as a TB diagnostic marker (Chen et al., 2018; Li, 2019). TRIM14 is a key regulator of the type I interferon response against *Mtb* infection (Hoffpauir et al., 2020). Second, *Mtb* might suppress cytokine signaling via RNF144B, a protein which plays an important role in activation of NF-*κ*B, cytokines and chemokines (Baek, Huang & Chang, 2016). RNF144B was suppressed in *Mtb*-infected macrophages, leading to increased intracellular survival of the pathogen (Banks et al., 2019). Therefore, the RNF144B-associated pathway might be a target for suppression during *Mtb* infection.

Different lineages of *Mtb* differ in virulence and in host-pathogen immune interactions. The lack of the TbD1 genome region in the more recent lineages (EA, EAI, EuA) is associated with a decrease of immune response level compared to the ancestral lineage (IO) (Portevin et al., 2011). Based on patient serum analysis and macrophage culture experiments, the IO lineage of *Mtb* induces higher levels of immune activation compared to the EA and EuA lineages (Amin et al., 2019; Portevin et al., 2011). Our study using primary cells in culture has also found that immune responses to the IO lineage differ from those to other lineages. MMP14 was highly expressed only in cells during active infection with the IO lineage. Following clearance, high expression levels of MMP14 were found in cells infected with the EA and EuA lineages as well as the H37Rv strain. MMP14 is a key protein required for leukocyte migration to the site of infection (Sathyamoorthy et al., 2015). The failure by the recent lineages of *Mtb* to promote production of MMP14 during active infection might benefit the pathogen by host immune suppression. DOCK11, DIDO1 and DMXL2 were only found in leukocytes during infection with the EuA lineage: these are associated with innate immunity and inflammation activation (Namekata et al., 2020). SPEN, DMXL1, APL17A, BCOR TTN, NEBL and C1orf174 were suppressed only in the EuA-lineage. Some of these proteins, such as BCOR, are related to apoptosis repression in *Mtb*-infected non-human primates (Mehra et al., 2010). The EuA lineage was found to induce high levels of cytokines and is known to have a high growth rate in human macrophages (Reiling et al., 2013). These responses might benefit the EuA lineage by upregulation of inflammation and suppression of apoptosis. Notably, the EA lineage (including the Beijing sub-lineage) is more strongly associated with increased transmissibility, relapse and drug resistance compared to other *Mtb* lineages (Burman et al., 2009; Feyisa et al., 2019; Glynn et al., 2002; Karmakar et al., 2019). In experiments using human monocyte-derived macrophages, strains of the EA lineage (including the Beijing sub-lineage) induced lower cytokine levels but exhibited higher replication rates than did other strains (Reiling et al., 2013). We found no protein uniquely expressed in the secretome of leukocytes infected with the EA lineage. However, some proteins (CPXM1, DCC, ZGPAT, TERF2 and TDRD7) were suppressed only in cells infected with EA lineages. Some of these proteins (e.g., CPXM1) are involved in protective immune responses against TB via interleukin-17, Th17 activation and cell homeostasis (Wareham et al., 2014). Therefore, the EA lineage might benefit from the suppression of host immune responses that promote pathogenesis. Similarly, no proteins were uniquely up-regulated or suppressed by the H37Rv strain. It is clear that immune responses differed when leukocytes were challenged with different lineages of *Mtb*. Such differences might explain the variability in vaccine efficacy among lineages of *M. tuberculosis* (Chae & Shin, 2018; Huang et al., 2020). Further comparisons among lineages, such as using intracellular proteomic analysis and animal-infection models, might clarify this situation.

Several immune-cell types participate in host-*Mtb* interactions. In this study, we demonstrated the role of each type during *Mtb* infection and following clearance. We did this by comparing the secretome with the specific database for each cell type. During active infection, the cell type-specific proteins reflected cell proliferation including macrophage cell growth and survival, cell-cycle progression of dendritic cell and B-cell cycle and neutrophil cell mitosis. Only proteins from regulatory T-cells were associated with an immune response against *Mtb* infection via proteins involved with antigen processing and presentation. Following pathogen clearance, the proteins secreted by each cell type differed from those found during the infection stage. Interferon-signaling proteins were commonly found from macrophages, dendritic cells and neutrophils. Other immune-response proteins were found following clearance, including those associated with macrophage phagocytosis, B-cell antigen presentation and T-cell antigen processing. However, proteins involved in cell proliferation (the key activity found during active infection) were found only in regulatory T-cells following clearance. These results might reflect suppression of the immune response caused by *Mtb.* A previous study suggested that immune suppression by *Mtb* occurred mainly in macrophages as the primary host (Zhai et al., 2019)*.* Our study suggests that immune suppression involves many cell types, as reflected by the immune-response proteins, especially those associated with the interferon-signaling pathway and antigen processing, found following clearance of *Mtb* from the host-cells. Trained immunity (which can involve many cell types) of these immune cells might be an alternative explanation (Lerm & Netea, 2016). It can be argued that the effect of BCG vaccination might mimic or confound the effect of trained immunity (Lalor et al., 2010). All our leukocyte donors were BCG-vaccinated and leukocytes from the same donors were compared in infection and control situations, thus controlling for the effect of BCG vaccination. It is possible that BCG vaccination could alter our secretome analysis result when compared to the unvaccinated condition. On the other hand, our participants, now around 30 years of age, received their BCG vaccine at birth. The protective effect of BCG is minimal in adults (Andersen & Doherty, 2005) and perhaps had no effect on our results.

A strength of using a mixed co-cultured cell population is that interaction among cell types can still occur, as would happen in intact immune-cell networks. We were then able to identify, using the IMEx interactome database, the proteins secreted by each type of immune cell. The specific response of individual cell types during infection and after clearance needs to be confirmed in the future. This could be done, for example, by excluding one type of immune cell at a time from the co-culture system and determining what proteins are missing. Uninfected cells activated with PMA and treated with INH and RIF were used as negative (background) controls. The secretome signals from the analysis were obtained after subtraction of the background control values and hence were not confounded by PMA or anti-TB drugs.

Limitations of this study should be noted. Heterogeneity in responses among individuals might be a confounding factor, hence we used leukocytes from three donors rather than a single donor. For logistical reasons, although we separately infected the leukocytes from each host in individual experiments, we pooled the extracellular proteins from all donors rather than analysing them individually. This could affect the results and interpretation. We did not determine the leukocyte population in each healthy donor: the proportions of WBC types were assumed to be in within the normal range. The leukocytes that we used were from human blood: leukocytes in lung tissues might exhibit different behaviours. Natural infection with *Mtb* is chronic, persisting for long periods: our *in-vitro* experiment was performed over only 5 days. Therefore, we used a low dose of PMA to activate the immune cells in an effort to mimic the natural activation of leukocytes (Sullivan et al., 2000). Thus primed, the cells should respond strongly and quickly to infection. However, PMA activation might skew this response. Previously, we have used a macrophage cell line (THP-1 cells) in an infection experiment and confirmed the results with western blotting (Kaewseekhao et al., 2015b). However, a single cell type cannot reflect the actual host immune environment. Here, we used primary leukocytes, a mixture of all immune cells, from human donors. Nonetheless, the host secretome was investigated in a cellular context which could distort the evaluation and interpretation of the actual host-pathogen interactions. We exposed our uninfected control cells to the same *Mtb*-killing drugs as the infected cells. Inclusion of an additional uninfected control group without drugs would have demonstrated whether use of these drugs influenced our results. Furthermore, the secretome of the clearance state could also reflect long-lasting or late responses to the infection. Due to budget constraints and the large number of proteins found, we did not confirm the results using western blotting as we have previously done (Kaewseekhao et al., 2015b). The high abundance of host protein interfered with detection of the secretome of the pathogen: we therefore did not analyse the pathogen secretome in our study.

## Conclusion

We demonstrated that the proteins secreted by *Mtb*-infected leukocytes differed according to the *Mtb* lineage involved. The ancestral lineage (IO lineage) had a greater ability to activate MMP14 (related to leukocyte migration) than did the more recent lineages (EA and EuA). During active infection, proteins from each type of immune cell were associated with cell proliferation whereas interferon-signaling and antigen-processing proteins were found after the pathogen was cleared from host cells. Immune response-related proteins from many immune cell types might be suppressed by *Mtb.* Our study has provided an improved insight into the host-pathogen interaction showing that immune response differ according to *Mtb* lineage.

##  Supplemental Information

10.7717/peerj.11565/supp-1 Supplemental Information 1 Genotypic characteristics of the three strains used, each representing one of the three main lineages of *M. tuberculosis*

10.7717/peerj.11565/supp-2 Supplemental Information 2 Classification of peptides according to qualitative or quantitative differences among *M. tuberculosis*-infected leukocytes under the various experimental conditions

10.7717/peerj.11565/supp-3 Supplemental Information 3 List of proteins, classified by experimental stage and *M. tuberculosis* lineage, detected from leukocytes used in the experiments

10.7717/peerj.11565/supp-4 Supplemental Information 4 Colony-forming unit (CFU) assays confirming the in-vitro clearance stage

10.7717/peerj.11565/supp-5 Supplemental Information 5 List of proteins expressed or suppressed during active infection and following clearance of *M. tuberculosis*

10.7717/peerj.11565/supp-6 Supplemental Information 6 List of top ten pathway associated with proteins expressed or suppressed during active infection and following clearance of *M. tuberculosis*

10.7717/peerj.11565/supp-7 Supplemental Information 7 List of proteins detected during active infection of leukocytes with strains representing several lineages of *M. tuberculosis*

10.7717/peerj.11565/supp-8 Supplemental Information 8 List of proteins suppressed during active infection of leukocytes with strains representing several lineages of *M. tuberculosis*

10.7717/peerj.11565/supp-9 Supplemental Information 9 Pathway analysis among immune-cell types during active infection and following clearance of *M. tuberculosis*

## Additional Information and Declarations

### Data Availability

The following information was supplied regarding data availability: The LC MS/MS raw data are available in the MassIVE database (MSV000084863): Supplementary data of secretome of Mtb infected cells. doi:10.25345/C5CD7Q.

## References

[ref-1] Amin M, Yanti B, Harapan H, Mertaniasih NM (2019). The role of *Mycobacterium tuberculosis* lineages on lung tissue damage and TNF-*α* level among tuberculosis patients, Indonesia. Clinical Epidemiology and Global Health.

[ref-2] Andersen P, Doherty TM (2005). The success and failure of BCG - implications for a novel tuberculosis vaccine. Nature Reviews Microbiology.

[ref-3] Baek S-H, Huang B, Chang HW (2016). RNF144b is a negative regulator in TLR2-mediated NF-kB activation. The Journal of Immunology.

[ref-4] Banks DA, Ahlbrand SE, Hughitt VK, Shah S, Mayer-Barber KD, Vogel SN, El-Sayed NM, Briken V (2019). Mycobacterium tuberculosis Inhibits Autocrine Type I IFN signaling to increase intracellular survival. The Journal of Immunology.

[ref-5] Brites D, Gagneux S (2012). Old and new selective pressures on *Mycobacterium tuberculosis*. Infection, Genetics and Evolution.

[ref-6] Burman WJ, Bliven EE, Cowan L, Bozeman L, Nahid P, Diem L, Vernon A (2009). Relapse associated with active disease caused by Beijing strain of *Mycobacterium tuberculosis*. Emerging Infectious Diseases.

[ref-7] Chae H, Shin SJ (2018). Importance of differential identification of *Mycobacterium tuberculosis* strains for understanding differences in their prevalence, treatment efficacy, and vaccine development. Journal of Microbiology.

[ref-8] Chai Q, Wang L, Liu CH, Ge B (2020). New insights into the evasion of host innate immunity by *Mycobacterium tuberculosis*. Cellular & Molecular Immunology.

[ref-9] Chen Y, Cao S, Sun Y, Li C (2018). Gene expression profiling of the TRIM protein family reveals potential biomarkers for indicating tuberculosis status. Microbial Pathogenesis.

[ref-10] Cooper AM (2009). Cell-mediated immune responses in tuberculosis. Annual Review of Immunology.

[ref-11] Coscolla M, Gagneux S (2014). Consequences of genomic diversity in *Mycobacterium tuberculosis*. Seminars in Immunology.

[ref-12] De Martino M, Lodi L, Galli L, Chiappini E (2019). Immune Response to *Mycobacterium tuberculosis*: a Narrative Review. Front Pediatr.

[ref-13] Feyisa SG, Abdurahman AA, Jimma W, Chaka EE, Kardan-Yamchi J, Kazemian H (2019). Resistance of *Mycobacterium tuberculosis* strains to Rifampicin: a systematic review and meta-analysis. Heliyon.

[ref-14] Gagneux S, DeRiemer K, Van T, Kato-Maeda M, De Jong BC, Narayanan S, Nicol M, Niemann S, Kremeri K, Gutierrez MC, Hilty M, Hopewell PC, Small PM (2006). Variable host-pathogen compatibility in *Mycobacterium tuberculosis*. Proceedings of the National Academy of Sciences of the United States of America.

[ref-15] Gideon HP, Phuah J, Junecko BA, Mattila JT (2019). Neutrophils express pro- and anti-inflammatory cytokines in granulomas from *Mycobacterium tuberculosis*-infected cynomolgus macaques. Mucosal Immunology.

[ref-16] Glynn JR, Whiteley J, Bifani PJ, Kremer K, Van Soolingen D (2002). Worldwide occurrence of Beijing/W strains of *Mycobacterium tuberculosis:* a systematic review. Emerging Infectious Diseases.

[ref-17] Hoffpauir CT, Bell SL, West KO, Jing T, Odio-Torres S, Cox JS, West AP, Li P, Patrick KL, Watson RO (2020). TRIM14 is a key regulator of the type I interferon response during *Mycobacterium tuberculosis* infection. bioRxiv Immunology.

[ref-18] Huang CC, Chu AL, Becerra MC, Galea JT, Calderon R, Contreras C, Yataco R, Zhang Z, Lecca L, Murray MB (2020). Mycobacterium tuberculosis Beijing lineage and risk for tuberculosis in child household contacts. Emerging Infectious Diseases.

[ref-19] Kaewseekhao B, Naranbhai V, Roytrakul S, Namwat W, Paemanee A, Lulitanond V, Chaiprasert A, Faksri K (2015a). Comparative proteomics of activated THP-1 cells infected with *Mycobacterium tuberculosis* identifies putative clearance biomarkers for tuberculosis treatment. PLOS ONE.

[ref-20] Kaewseekhao B, Naranbhai V, Roytrakul S, Namwat W, Paemanee A, Lulitanond V, Chaiprasert A, Faksri K (2015b). Comparative proteomics of activated THP-1 cells infected with *Mycobacterium tuberculosis* identifies putative clearance biomarkers for tuberculosis treatment. PLOS ONE.

[ref-21] Kaewseekhao B, Roytrakul S, Yingchutrakul Y, Salao K, Reechaipichitkul W, Faksri K (2020). Proteomic analysis of infected primary human leucocytes revealed PSTK as potential treatment-monitoring marker for active and latent tuberculosis. PLOS ONE.

[ref-22] Karmakar M, Trauer JM, Ascher DB, Denholm JT (2019). Hyper transmission of Beijing lineage *Mycobacterium tuberculosis:* systematic review and meta-analysis. Journal of Infection.

[ref-23] Lalor MK, Smith SG, Floyd S, Gorak-Stolinska P, Weir RE, Blitz R, Branson K, Fine PE, Dockrell HM (2010). Complex cytokine profiles induced by BCG vaccination in UK infants. Vaccine.

[ref-24] Lerm M, Netea MG (2016). Trained immunity: a new avenue for tuberculosis vaccine development. Journal of Internal Medicine: Blackwell Publishing Ltd.

[ref-25] Li C (2019). Gene Expression Profiling of TRIM Family in Individuals with Latent versus Active Tuberculosis and Reveals Potential Biomarkers for Diagnosis. The Journal of Immunology.

[ref-26] Li H, Wei S, Fang Y, Li M, Li X, Li Z, Zhang J, Zhu G, Li C, Bi L, Zhang G, Wang D, Zhang XE (2017). Quantitative proteomic analysis of host responses triggered by *Mycobacterium tuberculosis* infection in human macrophage cells. Acta Biochimica et Biophysica Sinica.

[ref-27] Lopez B, Aguilar D, Orozco H, Burger M, Espitia C, Ritacco V, Barrera L, Kremer K, Hernandez-Pando R, Huygen K, van Soolingen D (2003). A marked difference in pathogenesis and immune response induced by different *Mycobacterium tuberculosis* genotypes. Clinical and Experimental Immunology.

[ref-28] Mehra S, Pahar B, Dutta NK, Conerly CN, Philippi-Falkenstein K, Alvarez X, Kaushal D (2010). Transcriptional reprogramming in nonhuman primate (rhesus macaque) tuberculosis granulomas. PLOS ONE.

[ref-29] Monteiro-Maia R, Pinho RT (2014). Oral bacillus Calmette-Guerin vaccine against tuberculosis: why not?. Memórias do Instituto Oswaldo Cruz.

[ref-30] Namekata K, Guo X, Kimura A, Azuchi Y, Kitamura Y, Harada C, Harada T (2020). Roles of the DOCK-D family proteins in a mouse model of neuroinflammation. Journal of Biological Chemistry.

[ref-31] Orchard S, Kerrien S, Abbani S, Aranda B, Bhate J, Bidwell S, Bridge A, Briganti L, Brinkman F, Cesareni G, Chatr-Aryamontri A, Chautard E, Chen C, Dumousseau M, Goll J, Hancock R, Hannick LI, Jurisica I, Khadake J, Lynn DJ, Mahadevan U, Perfetto L, Raghunath A, Ricard-Blum S, Roechert B, Salwinski L, Stümpflen V, Tyers M, Uetz P, Xenarios I, Hermjakob H (2012). Protein Interaction data curation: the international Molecular Exchange (IMEx) consortium. Nature Methods: Nature Publishing Group.

[ref-32] Portevin D, Gagneux S, Comas I, Young D (2011). Human macrophage responses to clinical isolates from the *Mycobacterium tuberculosis* complex discriminate between ancient and modern lineages. PLOS Pathogens.

[ref-33] Reiling N, Homolka S, Walter K, Brandenburg J, Niwinski L, Ernst M, Herzmann C, Lange C, Diel R, Ehlers S, Niemann S (2013). Clade-specific virulence patterns of *Mycobacterium tuberculosis* complex strains in human primary macrophages and aerogenically infected mice. mBio.

[ref-34] Sarkar R, Lenders L, Wilkinson KA, Wilkinson RJ, Nicol MP (2012). Modern lineages of *Mycobacterium tuberculosis* exhibit lineage-specific patterns of growth and cytokine induction in human monocyte-derived macrophages. PLOS ONE.

[ref-35] Sathyamoorthy T, Tezera LB, Walker NF, Brilha S, Saraiva L, Mauri FA, Wilkinson RJ, Friedland JS, Elkington PT (2015). Membrane type 1 matrix metalloproteinase regulates monocyte migration and collagen destruction in tuberculosis. The Journal of Immunology.

[ref-36] Subbian S, Bandyopadhyay N, Tsenova L, O’Brien P, Khetani V, Kushner NL, Peixoto B, Soteropoulos P, Bader JS, Karakousis PC, Fallows D, Kaplan G (2013). Early innate immunity determines outcome of *Mycobacterium tuberculosis* pulmonary infection in rabbits. Cell Communication and Signaling.

[ref-37] Sullivan KE, Cutilli J, Piliero LM, Ghavimi-Alagha D, Starr SE, Campbell DE, Douglas SD (2000). Measurement of cytokine secretion, intracellular protein expression, and mRNA in resting and stimulated peripheral blood mononuclear cells. Clinical and Diagnostic Laboratory Immunology.

[ref-38] Tientcheu LD, Koch A, Ndengane M, Andoseh G, Kampmann B, Wilkinson RJ (2017). Immunological consequences of strain variation within the *Mycobacterium tuberculosis* complex. European Journal of Immunology.

[ref-39] Tram TTB, Nhung HN, Vijay S, Hai HT, Thu DDA, Ha VTN, Dinh TD, Ashton PM, Hanh NT, Phu NH, Thwaites GE, Thuong NTT (2018). Virulence of *Mycobacterium tuberculosis* clinical isolates is associated with sputum pre-treatment bacterial load, lineage, survival in macrophages, and cytokine response. Frontiers in Cellular and Infection Microbiology.

[ref-40] Van Soolingen D, Dormans J, Burger M, Aguilar D, Hernandez-Pando R, Kremer K, Roholl P, Arend SM (2004). Correlation of virulence, lung pathology, bacterial load and delayed type hypersensitivity responses after infection with different *Mycobacterium tuberculosis* genotypes in a BALB/c mouse model. Clinical and Experimental Immunology.

[ref-41] Vandenbon A, Dinh VH, Mikami N, Kitagawa Y, Teraguchi S, Ohkura N, Sakaguchi S (2016). Immuno-Navigator, a batch-corrected coexpression database, reveals cell type-specific gene networks in the immune system. Proceedings of the National Academy of Sciences of the United States of America.

[ref-42] Wareham AS, Tree JA, Marsh PD, Butcher PD, Dennis M, Sharpe SA (2014). Evidence for a role for interleukin-17, Th17 cells and iron homeostasis in protective immunity against tuberculosis in cynomolgus macaques. PLOS ONE.

[ref-43] Waterborg JH, Walker JM (2009). The lowry method for protein quantitation. The Protein Protocols Handbook. Springer Protocols Handbooks.

[ref-44] WHO (2019). Global tuberculosis report 2019.

[ref-45] Xia J, Benner MJ, Hancock REW (2014). NetworkAnalyst - integrative approaches for protein-protein interaction network analysis and visual exploration. Nucleic Acids Research.

[ref-46] Yang Q, Xu Q, Chen Q, Li J, Zhang M, Cai Y, Liu H, Zhou Y, Deng G, Deng Q, Zhou B, Kornfeld H, Chen X (2015). Discriminating active tuberculosis from latent tuberculosis infection by flow cytometric measurement of CD161-expressing T cells. Scientific Reports.

[ref-47] Yuan C-H, Zhang S, Xiang F, Gong H, Wang Q, Chen Y, Luo W (2019). Secreted Rv1768 from RD14 of *Mycobacterium tuberculosis* activates macrophages and induces a strong IFN- *γ*-releasing of CD4+ T cells. Frontiers in Cellular and Infection Microbiology.

[ref-48] Zhai W, Wu F, Zhang Y, Fu Y, Liu Z (2019). The immune escape mechanisms of *Mycobacterium tuberculosis*. International Journal of Molecular Sciences.

